# Frequency-specific resting-state EEG modulation during eyes-closed and eyes-open transitions in female soccer players

**DOI:** 10.3389/fpsyg.2026.1907876

**Published:** 2026-09-15

**Authors:** Zhi Gu, Hao-Wei Ma, Bo Gong

**Affiliations:** 1 School of Athletic Performance, Shanghai University of Sport, Shanghai, China; 2 School of Physical Education, Henan University, Kaifeng, China

**Keywords:** electroencephalography, neural modulation, resting-state EEG, soccer players, sport psychology

## Abstract

This study aimed to investigate differences in resting-state electroencephalographic (EEG) activity between female soccer players and female university students under eyes-open (EO) and eyes-closed (EC) conditions. A total of 25 female soccer players and 20 female university students participated in the study. Resting-state EEG signals were recorded using a 32-channel EEG system during 3 min of EO and 3 min of EC conditions. Absolute power in the delta (0.5–4 Hz), theta (4–8 Hz), alpha (8–13 Hz), and beta (13–30 Hz) frequency bands was calculated using Fast Fourier Transform (FFT). Electrodes were grouped into four regions of interest (ROIs): frontal, central, parietal, and occipital regions. A three-way repeated-measures analysis of variance (Group × Condition × ROI) and difference-score analysis (Δ = EC – EO) were conducted to examine group differences. The results revealed significant main effects of Condition and ROI across all frequency bands (all *p* < 0.01), indicating that the EC–EO transition reliably modulated resting-state EEG activity. No significant group-related effects were observed in the alpha or theta bands (all *p* > 0.05). In contrast, a significant Condition × ROI × Group interaction was found in the beta band (*p* < 0.05), whereas the delta band exhibited significant Condition × Group and Condition × ROI × Group interactions (both *p* < 0.05). Furthermore, difference-score analyses revealed exploratory differences in EC–EO modulation between groups in the beta and delta bands before correction for multiple comparisons; however, these effects did not remain significant after FDR correction. In conclusion, resting-state EEG modulation patterns differed between female soccer players and female university students in a frequency- and region-dependent manner, particularly involving the beta and delta bands. In contrast, alpha and theta activity mainly reflected fundamental physiological responses to EO and EC conditions and showed no apparent sport-specific differences. These findings suggest that long-term soccer participation may be associated with differences in resting-state neural regulatory patterns involving oscillatory processes previously implicated in visual information processing and sensorimotor regulation, although the functional significance of these patterns requires further validation. The present study provides new empirical evidence for understanding sport-related neural adaptations in open-skill sports.

## 1 Introduction

In the field of sport neuroscience, resting-state electroencephalography (EEG) has been widely used to characterize intrinsic neural activity and assess the brain's capacity to adapt to environmental changes (Barry et al., 2007; Cohen, 2014; Raichle, 2015). Among resting-state paradigms, differences in EEG activity between eyes-open (EO) and eyes-closed (EC) conditions are considered an important model for investigating the transition from internally oriented information processing to externally directed perceptual processing (Petro et al., 2022; Han et al., 2023; Ingram et al., 2024). Previous studies have demonstrated that the EC–EO transition not only induces systematic changes in EEG power but also leads to substantial reorganization of spatial activity patterns, particularly within posterior visual-related cortical regions (Li et al., 2009; Petro et al., 2022). This phenomenon is generally interpreted as a process of cortical network reconfiguration triggered by the introduction of visual input, involving key mechanisms such as attentional resource redistribution and sensory information integration. Consequently, the EC–EO paradigm provides a low-demand and highly reproducible experimental framework for examining neural regulatory capacity across different populations.

From a functional perspective, distinct EEG oscillatory frequency bands are associated with relatively specific neural processes. Although the delta band has traditionally been classified as a slow-wave oscillation, accumulating evidence suggests that it also contributes to attentional resource allocation and environmental monitoring during wakefulness (Knyazev, 2012; Başar, 2013; Harmony, 2013). Theta oscillations are primarily associated with cognitive control and working memory processes (Cavanagh and Frank, 2014; Cooper et al., 2019; Riddle et al., 2020). The alpha band is commonly regarded as an indicator of visual inhibition and internally directed attention, showing increased power during EC and suppression during EO conditions (Klimesch, 2012; Han et al., 2023). Beta activity is closely linked to active information processing, sensorimotor integration, and higher-order cognitive control (Kilavik et al., 2013; Spitzer and Haegens, 2017). Therefore, the combined analysis of multiple frequency bands provides a comprehensive approach for characterizing the dynamic neural regulation underlying state transitions from different functional perspectives.

In athletic populations, long-term sport-specific training is believed to reshape functional brain organization through experience-dependent plasticity (Hötting and Röder, 2013). A substantial body of behavioral and neuroimaging research has shown that athletes generally outperform non-athletes in visual attention, spatial perception, and decision-making performance (Vestberg et al., 2017; Ishak et al., 2023; Imperiali et al., 2025; Mahlangu et al., 2025; Ren et al., 2025). However, the manifestation of these advantages in resting-state EEG activity, particularly during the EC–EO transition, remains poorly understood and lacks consistent empirical evidence. Several EEG studies have reported sport-specific differences in alpha-, beta-, and slow-wave power distributions among athletes, which may reflect long-term adaptations in the processing of environmental information (Fang et al., 2022; Ramyarangsi et al., 2024; Zhang et al., 2025). Nevertheless, these differences often appear to be frequency- and region-dependent rather than uniformly distributed across the entire brain. Furthermore, distinct neural regulation patterns have been observed across different sport types, with open-skill and closed-skill sports exhibiting divergent neural adaptation characteristics (Veliks et al., 2024).

Soccer is a representative open-skill sport in which performance depends heavily on the continuous monitoring of dynamic environmental information and rapid behavioral responses (Lai et al., 2024; Triggs et al., 2025). During competition, players must constantly integrate visual information, maintain spatial awareness, and execute decisions and motor actions within extremely short time frames. These processes require highly coordinated interactions among visual, attentional, and sensorimotor networks (Klatt and Smeeton, 2022; Schumacher et al., 2024). Therefore, it can be hypothesized that soccer players may exhibit different patterns of neural regulation when transitioning from an internally oriented resting state (EC) to an externally oriented environmental monitoring state (EO). Previous studies have also suggested that soccer players may demonstrate distinct resting-state EEG patterns, particularly within parietal and occipital regions associated with visuospatial processing; however, the available evidence remains limited and findings have been inconsistent (Ramyarangsi et al., 2024; Rubio-Morales et al., 2025).

Importantly, two major gaps remain in the existing literature. First, most studies investigating EEG characteristics in athletes have focused on male or mixed-sex samples, whereas research involving female athletes remains relatively scarce. Given that sex-related differences may influence EEG oscillatory activity and its modulation, findings derived from male-dominated samples may not be directly generalizable to female athletes. Second, although previous studies have compared EEG characteristics across different sports and training levels, systematic investigations of resting-state EC–EO transitions in soccer players relative to non-athletic controls remain limited. In particular, few studies have examined these differences using an integrated multi-frequency and multi-regional approach. Consequently, it remains unclear whether long-term soccer participation is associated with distinct neural regulatory patterns during basic state transitions and whether such patterns exhibit frequency- and region-specific characteristics.

Against this background, the present study compared female soccer players and female university students with respect to EEG power across different brain regions (frontal, central, parietal, and occipital) and frequency bands (delta, theta, alpha, and beta) under resting-state EC and EO conditions. Using a three-way repeated-measures analysis of variance and difference-score analysis (Δ = EC – EO), we examined the interaction effects among group, condition, and region of interest (ROI), and further explored the spatial distribution of group differences. This study aims to address the current gap in knowledge regarding resting-state neural regulation in female soccer players and to provide new empirical evidence regarding neural characteristics associated with open-skill sports expertise.

## 2 Methods

### 2.1 Participants

A total of 45 participants were recruited for this study, including 25 female soccer players with systematic training backgrounds assigned to the experimental group and 20 female university students assigned to the control group. All participants were between 18 and 23 years of age, right-handed, and had normal or corrected-to-normal vision. The soccer players had accumulated at least 10 years of systematic soccer training experience and had extensive competitive experience. All were members of teams competing in the China Taiping CFA Women's Football League, the second-tier national women's soccer league in China. Among the 25 soccer players, 2 were goalkeepers, 9 were defenders, 8 were midfielders, and 6 were forwards. Due to retrospective data availability limitations, detailed quantification of current weekly training volume and intensity was not available; therefore, these factors were considered as potential uncontrolled variables in the present study. The control group consisted of female university students with no history of systematic training in team ball sports.

The demographic characteristics of the participants are presented in Table 1. No significant between-group differences were observed in age, height, body weight, or BMI (all p > 0.05). Sample size estimation was conducted based on previous related research. According to a previously reported effect size of η*p*^2^ = 0.20, the corresponding Cohen's *f* was calculated as 0.50 (Cave and Barry, 2021). Assuming a significance level of α = 0.05 and a statistical power of 1 – β = 0.80, the minimum required total sample size was estimated to be 34 participants. The final sample consisted of 45 participants, exceeding the minimum requirement. Although the sample sizes of the two groups were not identical, the mixed-design analysis of variance employed in this study is capable of accommodating unequal group sizes. Written informed consent was obtained from all participants prior to participation. The study protocol was approved by the Ethics Committee of Shanghai University of Sport (Approval No. 10277RT250306) and was conducted in accordance with the principles of the Declaration of Helsinki.

**Table 1:** Participant characteristics of the control and soccer groups.

Variable	Con	Soc
Age (yrs)	21.6 ± 0.78	21.7 ± 1.3
Height (cm)	163.4 ± 2.66	166.48 ± 4.92
Body weight (kg)	55.6 ± 3.83	57.56 ± 5.75
BMI	20.83 ± 1.37	20.74 ± 1.48

Data are presented as mean ± standard deviation (SD).

Con, control group; Soc, soccer group.

BMI, body mass index.

Height is expressed in centimeters (cm), body weight in kilograms (kg), and BMI in kg/m^2^.

### 2.2 Experimental procedure

To minimize the influence of external factors on resting-state EEG signals, all participants were instructed to refrain from consuming caffeinated beverages for 24 h and alcohol for 48 h prior to testing, and to obtain sufficient sleep on the night before the experiment. Participants were also asked to avoid prolonged screen exposure before testing to reduce the potential influence of visual fatigue on EEG activity. All experimental sessions were conducted at the same time of day to minimize potential circadian effects.

The experiment was performed in a quiet laboratory with stable lighting conditions (approximately 100–150 lx) and a controlled ambient temperature of 22–24 °C. Upon arrival, participants rested quietly for 2 min to acclimate to the testing environment before EEG recording commenced. All participants were seated comfortably in an upright position during EEG recording, and the same posture was maintained throughout both eyes-open (EO) and eyes-closed (EC) conditions.

Resting-state EEG activity was recorded under two conditions: eyes open (EO) and eyes closed (EC). Each condition lasted 3 min. The order of conditions was fixed, with the EO condition always preceding the EC condition, and the same sequence was applied to all participants. During the EO condition, participants were instructed to maintain visual fixation on a target positioned directly in front of them while minimizing eye movements and body movements throughout the recording period. During the EC condition, participants were instructed to remain relaxed with their eyes closed while avoiding unnecessary movements.

### 2.3 Electroencephalography (EEG) assessments

EEG signals were recorded using a 32-channel EEG system with Ag/AgCl electrodes positioned according to the International 10–20 System. During data acquisition, bilateral mastoid electrodes served as the reference electrodes. Electrooculogram (EOG) signals were simultaneously recorded to monitor ocular artifacts.

Prior to EEG recording, the scalp areas corresponding to electrode locations were cleaned, and conductive gel was applied to reduce electrode impedance. Impedance was maintained below 5 kΩ for all channels throughout the recording session. Participants were seated comfortably and instructed to minimize body and head movements during data acquisition to ensure high-quality EEG recordings.

### 2.4 EEG data acquisition

EEG signals were sampled at 500 Hz. Offline preprocessing was performed using EEGLAB (Version 2021.0). Raw EEG data were first band-pass filtered between 0.1 and 30 Hz and subsequently notch filtered between 49 and 51 Hz to remove 50 Hz power-line interference. Independent component analysis (ICA) was then applied to identify and remove ocular and other stereotypical artifacts. Independent components associated with ocular activity were identified based on their scalp topographies, time-course characteristics, and correlation with recorded electrooculographic (EOG) signals, and were removed before reconstructing the continuous EEG data.

Continuous resting-state EEG data from the eyes-open (EO) and eyes-closed (EC) conditions were subsequently extracted, and 180 s of data were retained for each condition. The continuous data were segmented into consecutive 2-s epochs for subsequent analyses. Epochs with amplitudes exceeding ±100 μV were automatically rejected to further minimize the influence of residual movement artifacts and other non-physiological noise.

Prior to spectral analysis, the TP9 and TP10 channels were removed because these electrodes are not part of the standard International 10–20 system and were not included in the predefined regional analyses. The remaining 29 EEG channels were re-referenced using an average reference. Absolute power (μ*V*^2^) for each frequency band was subsequently calculated using Fast Fourier Transform (FFT). For regional analyses, electrodes were grouped into predefined regions of interest (ROIs), and mean power values were calculated by averaging the electrodes included within each ROI. Frequency bands were defined as delta (0.5–4 Hz), theta (4–8 Hz), alpha (8–13 Hz), and beta (13–30 Hz).

Based on the spatial distribution of electrodes according to the International 10–20 System and their functional anatomical characteristics, electrodes were grouped into four regions of interest (ROIs): frontal, central, parietal, and occipital regions. The frontal ROI comprised F3, F4, F7, F8, and Fz electrodes; the central ROI comprised C3, C4, and Cz electrodes; the parietal ROI comprised P3, P4, and Pz electrodes; and the occipital ROI comprised O1 and O2 electrodes. Mean power values were calculated by averaging the electrodes within each ROI and were used for subsequent statistical analyses. The ROI definition was based on the conventional 10–20 electrode placement system and was consistent with previous resting-state EEG studies investigating regional spectral characteristics.

### 2.5 Statistical analysis

All statistical analyses were performed using SPSS Statistics (Version 28.0; IBM Corp., Armonk, NY, USA). Data normality was assessed using the Shapiro–Wilk test.

For each frequency band, a three-way repeated-measures analysis of variance (ANOVA) was conducted, with Group (soccer players vs. controls) as the between-subject factor and Condition (EC vs. EO) and Region of Interest (ROI: frontal, central, parietal, and occipital) as within-subject factors. Frequency bands were analyzed separately because each oscillatory band represents distinct neurophysiological processes and may exhibit different patterns of modulation during resting-state transitions. Given the exploratory nature of examining frequency-specific modulation patterns, the results of frequency-specific ANOVA models were interpreted cautiously, particularly for interaction effects involving Group. Main effects and interaction effects were evaluated, and corresponding *F* values, *p* values, and partial eta-squared (η*p*^2^) effect sizes were reported. When the assumption of sphericity was violated, Greenhouse–Geisser corrections were applied. Significant interactions were further examined using Bonferroni-adjusted *post hoc* comparisons.

To further characterize the magnitude of EC–EO modulation, difference scores were calculated for each participant and frequency band (Δ = EC – EO). Independent-samples *t*-tests were subsequently performed to compare Δ values between groups across ROIs and frequency bands. Because multiple exploratory comparisons were conducted across frequency bands and ROIs, false discovery rate (FDR) correction was applied to control the potential inflation of type I error. Both uncorrected *p* values and FDR-adjusted q values were reported. Corresponding *t* values, Cohen's *d* effect sizes, and 95% confidence intervals (CIs) of the effect sizes were calculated to quantify the magnitude and precision of between-group differences. Effect sizes were interpreted together with their confidence intervals to provide additional information beyond statistical significance. Homogeneity of variance was assessed prior to group comparisons, and Welch's correction was applied when the assumption of equal variances was violated. All statistical tests were two-tailed, and statistical significance was set at *p* < 0.05.

## 3 Results

### 3.1 Descriptive statistics

Descriptive statistics for EEG power across conditions and regions of interest (ROIs) are presented in Table 2. The topographical distribution of EEG power across frequency bands and conditions is illustrated in Figure 1. Overall, EEG power varied across conditions and ROIs, with higher power generally observed during the EC condition than during the EO condition.

**Table 2:** Descriptive statistics of EEG power (mean ± SD) across conditions, groups, frequency bands, and ROIs.

Frequency	ROI	EC		EO	
		Con	Soc	Con	Soc
Delta	Frontal	37.11 ± 1.98	37.70 ± 2.31	36.93 ± 2.87	35.97 ± 1.92
	Central	33.17 ± 1.32	33.04 ± 1.90	32.32 ± 1.78	31.86 ± 1.33
	Parietal	35.24 ± 1.57	36.49 ± 2.52	35.43 ± 1.99	34.94 ± 1.69
	Occipital	36.89 ± 1.53	37.76 ± 2.52	37.13 ± 2.54	36.33 ± 2.01
Theta	Frontal	31.19 ± 2.17	32.65 ± 2.15	30.92 ± 2.23	31.26 ± 2.53
	Central	28.75 ± 2.13	29.89 ± 3.17	27.55 ± 2.11	28.52 ± 2.52
	Parietal	30.81 ± 2.50	32.30 ± 3.98	29.44 ± 2.27	30.10 ± 2.67
	Occipital	32.86 ± 2.32	33.82 ± 3.68	31.24 ± 1.97	31.74 ± 2.60
Alpha	Frontal	34.69 ± 3.23	34.72 ± 4.30	30.12 ± 2.99	29.61 ± 3.89
	Central	33.01 ± 3.18	32.97 ± 4.10	28.93 ± 3.58	28.56 ± 4.56
	Parietal	38.49 ± 4.11	38.21 ± 5.09	32.50 ± 3.81	31.59 ± 5.05
	Occipital	41.02 ± 2.80	39.98 ± 4.27	34.21 ± 2.93	32.53 ± 4.10
Beta	Frontal	24.83 ± 2.37	25.43 ± 1.58	26.93 ± 3.64	25.89 ± 2.70
	Central	22.96 ± 2.73	23.85 ± 2.25	22.31 ± 2.95	23.15 ± 1.98
	Parietal	26.32 ± 2.98	27.35 ± 2.40	24.37 ± 2.87	24.96 ± 2.34
	Occipital	30.43 ± 2.76	30.88 ± 2.97	29.15 ± 2.79	29.06 ± 3.74

Values are presented as mean ± standard deviation (SD).

EC, eyes closed; EO, eyes open; Con, control group; Soc, soccer group; ROI, region of interest.

EEG power is expressed in μV^2^. Frequency bands include delta (0.5–4 Hz), theta (4–8 Hz), alpha (8–13 Hz), and beta (13–30 Hz).

**Figure 1:**
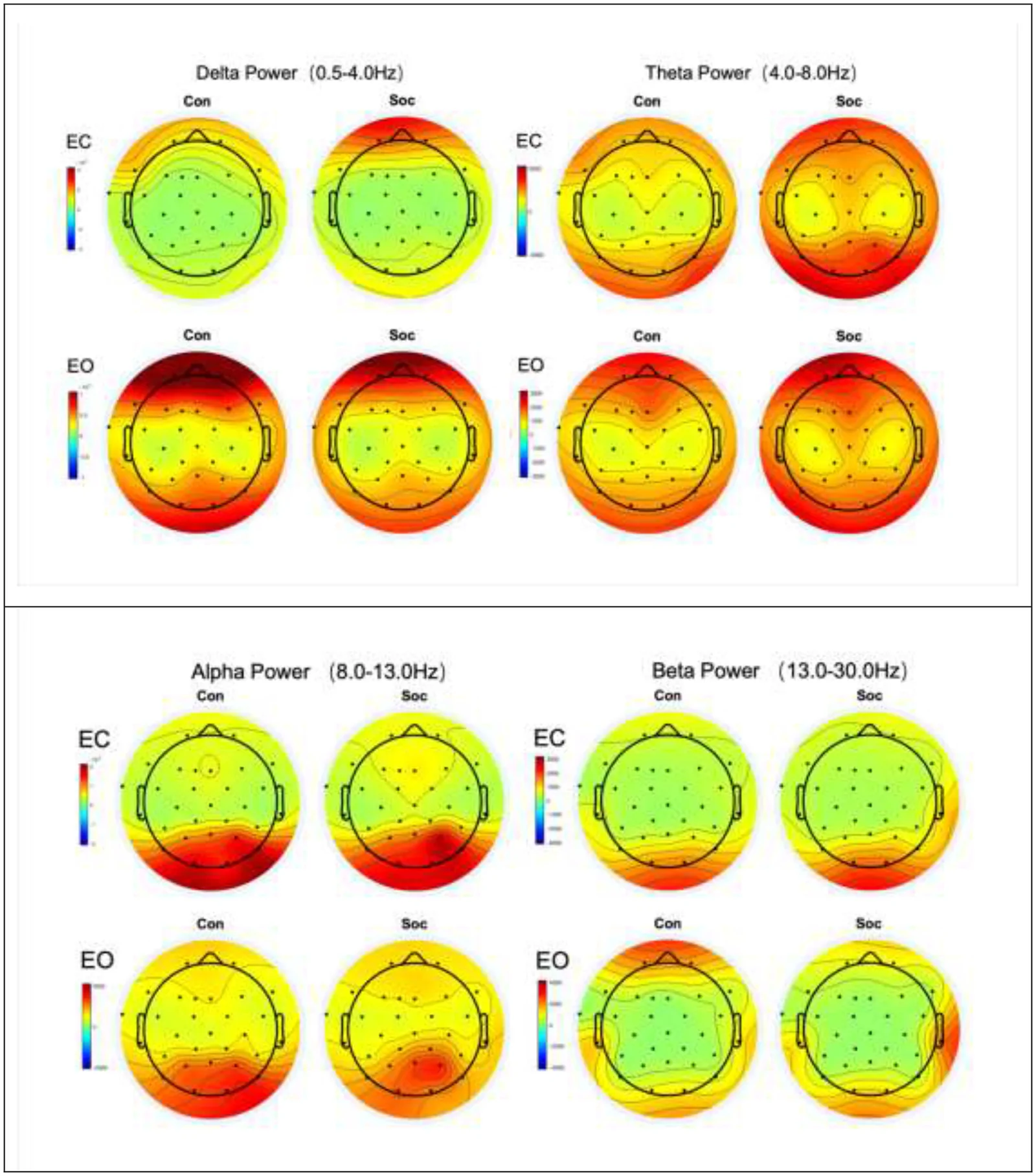
Topographical distribution of EEG power across delta (0.5–4 Hz), theta (4–8 Hz), alpha (8–13 Hz), and beta (13–30 Hz) frequency bands during eyes-closed (EC) and eyes-open (EO) conditions in control and soccer groups.

### 3.2 Group differences in EC–EO modulation (Δ = EC – EO)

As shown in Table 3, after FDR correction for multiple comparisons, no significant between-group differences were observed in EEG power modulation (Δ = EC – EO) across any frequency band or ROI (all *q* > 0.05). Before correction, uncorrected analyses indicated potential differences in the beta and delta bands. Specifically, the beta band showed an uncorrected difference in the frontal region (*p* = 0.024, Cohen's *d* = −0.701), whereas the delta band showed uncorrected differences in the parietal (*p* = 0.006, Cohen's d = −0.859) and occipital regions (*p* = 0.035, Cohen's *d* = −0.655). However, these effects did not remain significant after FDR correction. Although none of the exploratory comparisons survived FDR correction, the effect size estimates indicated moderate differences in several regional comparisons, particularly in the delta parietal region (Cohen's *d* = −0.859, 95% CI: −1.474 to −0.244) and beta frontal region (Cohen's *d* = −0.701, 95% CI: −1.307 to −0.095). However, the confidence intervals should be interpreted cautiously because they reflect uncertainty associated with the relatively limited sample size.

**Table 3:** Exploratory between-group comparisons of EEG power modulation (Δ = EC – EO) with FDR correction and effect size estimates.

Frequency	ROI	Con	Soc	*t*(df)	*p*	FDR *q*	Cohen's *d*	95% CI
delta	Frontal	0.17 ± 3.12	1.74 ± 2.86	−1.751	0.087	0.278	−0.525	−1.123 to 0.073
	Central	0.86 ± 1.21	1.19 ± 1.04	−0.981	0.332	0.59	−0.294	−0.885 to 0.297
	Parietal	−0.20 ± 2.16	1.55 ± 1.93	−2.864	0.006	0.096	−0.859	−1.474 to −0.244
	Occipital	−0.24 ± 2.92	1.43 ± 2.24	−2.182	0.035	0.187	−0.655	−1.259 to −0.051
theta	Frontal	0.27 ± 2.19	1.39 ± 1.86	−1.862	0.069	0.276	−0.559	−1.159 to 0.041
	Central	1.19 ± 0.97	1.37 ± 1.74	−0.424	0.674	0.719	−0.127	−0.716 to 0.462
	Parietal	1.37 ± 1.96	2.20 ± 2.34	−1.265	0.213	0.568	−0.38	−0.973 to 0.213
	Occipital	1.62 ± 2.35	2.08 ± 2.26	−0.671	0.506	0.68	−0.201	−0.791 to 0.389
alpha	Frontal	4.60 ± 2.35	5.11 ± 2.99	−0.664	0.51	0.68	−0.199	−0.789 to 0.391
	Central	4.08 ± 2.28	4.42 ± 2.47	−0.472	0.64	0.719	−0.141	−0.730 to 0.448
	Parietal	5.99 ± 2.85	6.63 ± 3.15	−0.709	0.482	0.68	−0.213	−0.803 to 0.377
	Occipital	6.81 ± 2.97	7.45 ± 4.00	−0.594	0.556	0.684	−0.178	−0.767 to 0.411
beta	Frontal	−2.09 ± 2.61	−0.46 ± 2.09	−2.338	0.024	0.187	−0.701	−1.307 to −0.095
	Central	0.65 ± 1.02	0.70 ± 1.38	−0.114	0.91	0.91	−0.034	−0.622 to 0.554
	Parietal	1.95 ± 1.37	2.38 ± 1.46	−1.005	0.32	0.59	−0.302	−0.893 to 0.289
	Occipital	1.29 ± 1.39	1.82 ± 1.93	−1.038	0.305	0.59	−0.311	−0.903 to 0.281

Values are presented as mean ± standard deviation (SD).

Δ = EC – EO represents the difference in EEG power between the eyes-closed and eyes-open conditions.

Con, control group; Soc, soccer group; ROI, region of interest.

EEG power is expressed in μV^2^. FDR q values were calculated using the Benjamini–Hochberg procedure to correct for multiple comparisons across the 16 between-group comparisons. Statistical significance after FDR correction was defined as q < 0.05. Cohen's d values and corresponding 95% confidence intervals (CIs) were provided to describe the magnitude and precision of between-group differences.

### 3.3 Repeated-measures ANOVA

As shown in Table 4, significant main effects of Condition (all *p* < 0.01) and ROI (all *p* < 0.001) were observed across all frequency bands. For the alpha and theta bands, significant Condition × ROI interactions were found (both *p* < 0.001), whereas no significant Group-related effects were observed (all *p* > 0.05). In the beta band, in addition to a significant Condition × ROI interaction (*p* < 0.001), a significant three-way interaction among Condition, ROI, and Group was observed (*F*(3,129) = 3.109, *p* = 0.029, η*p*^2^ = 0.067). In the delta band, no significant Condition × ROI interaction was observed.

**Table 4:** Results of repeated-measures ANOVA for EEG power across frequency bands (group × condition × ROI).

Effect	*F*	df	*p*	*ηp* ^2^
delta ROI				
Condition	7.708	1, 43	0.008	0.152
Condition × group	5.158	1, 43	0.028	0.107
ROI	166.283	3, 129	< 0.001	0.795
ROI × group	0.864	3, 129	0.462	0.02
Condition × ROI	1.034	3, 129	0.38	0.023
Condition × ROI × group	2.688	3, 129	0.049	0.059
theta ROI				
Condition	27.444	1, 43	< 0.001	0.39
Condition × group	1.406	1, 43	0.242	0.032
ROI	105.157	3, 129	< 0.001	0.71
ROI × group	0.262	3, 129	0.853	0.006
Condition × ROI	10.617	3, 129	< 0.001	0.198
Condition × ROI × group	1.981	3, 129	0.12	0.044
alpha ROI				
Condition	185.742	1, 43	< 0.001	0.812
Condition × group	0.427	1, 43	0.517	0.01
ROI	153.435	3, 129	< 0.001	0.781
ROI × group	1.46	3, 129	0.229	0.033
Condition × ROI	49.262	3, 129	< 0.001	0.534
Condition × ROI × group	0.143	3, 129	0.934	0.003
beta ROI				
Condition	15.688	1, 43	< 0.001	0.267
Condition × group	2.81	1, 43	0.101	0.061
ROI	100.796	3, 129	< 0.001	0.701
ROI × group	0.867	3, 129	0.46	0.02
Condition × ROI	59.852	3, 129	< 0.001	0.582
Condition × ROI × group	3.109	3, 129	0.029	0.067

Results are presented as F values with corresponding degrees of freedom (df), p values, and partial eta squared (ηp^2^). Group (soccer vs. control) was included as a between-subject factor, and Condition (eyes closed vs. eyes open) and ROI (frontal, central, parietal, occipital) were included as within-subject factors. Analyses were conducted separately for each frequency band. Significant effects are indicated at p < 0.05.

However, significant Condition × Group and Condition × ROI × Group interactions were found. Furthermore, significant Condition × Group (*F*(1,43) = 5.158, *p* = 0.028, η*p*^2^ = 0.107) and Condition × ROI × Group interactions (*F*(3,129) = 2.688, *p* = 0.049, η*p*^2^ = 0.059) were observed.

### 3.4 *Post hoc* analysis (estimated marginal means)

As shown in Table 5 in the beta band, significant differences between the EC and EO conditions were observed only in the soccer group (*p* < 0.001), whereas no significant difference was found in the control group (*p* = 0.133). Although the overall condition effect was not significant in the control group, ROI-specific analyses revealed localized EC–EO differences across all ROIs. The regional patterns of beta-band activity under EO and EC conditions are illustrated in Figure 2.

**Table 5:** *Post hoc* comparisons of condition effects (EC vs. EO) within each group and across regions of interest.

Condition effect within each group				
Frequency	Group	ROI	Mean diff (EC–EO)	*p*
Delta	Control	Overall	0.148	0.736
Delta	Soccer	Overall	1.477	< 0.001
Beta	Control	Overall	0.45	0.133
Beta	Soccer	Overall	1.109	< 0.001
ROI-specific effects				
Delta	Control	Central	0.857	0.001
Delta	Soccer	Frontal	1.736	0.006
Delta	Soccer	Central	1.187	< 0.001
Delta	Soccer	Parietal	1.553	< 0.001
Delta	Soccer	Occipital	1.431	0.008
Beta	Control	Frontal	−2.094	< 0.001
Beta	Control	Central	0.653	0.022
Beta	Control	Parietal	1.953	< 0.001
Beta	Control	Occipital	1.285	0.002
Beta	Soccer	Central	0.695	0.007
Beta	Soccer	Parietal	2.381	< 0.001
Beta	Soccer	Occipital	1.819	< 0.001

Mean differences represent EC – EO (eyes closed minus eyes open).

Positive values indicate higher EEG power in the EC condition.

Con, control group; Soc, soccer group; ROI, region of interest.

*Post hoc* comparisons were conducted using estimated marginal means (EMMEANS) with Bonferroni correction for multiple comparisons. Statistical significance was set at p < 0.05.

**Figure 2:**
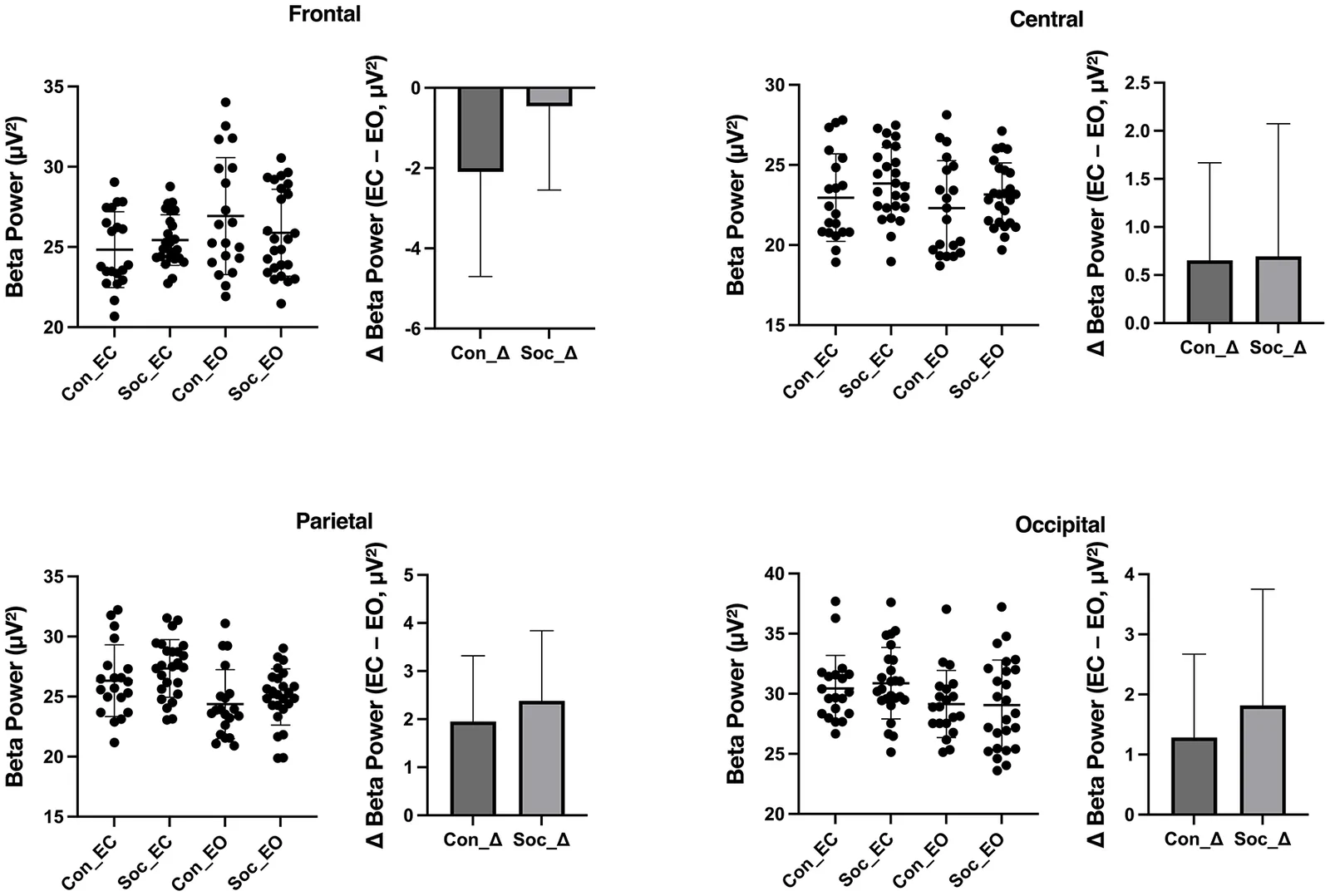
Regional patterns of beta-band EEG power during eyes-closed (EC) and eyes-open (EO) conditions and corresponding EC–EO modulation (Δ = EC – EO) in control and soccer groups.

In the delta band, the soccer group exhibited significant EC–EO differences across all ROIs (all *p* < 0.01), whereas the control group showed a significant difference only in the central region (*p* = 0.001). The corresponding regional patterns of delta-band activity are presented in Figure 3.

**Figure 3:**
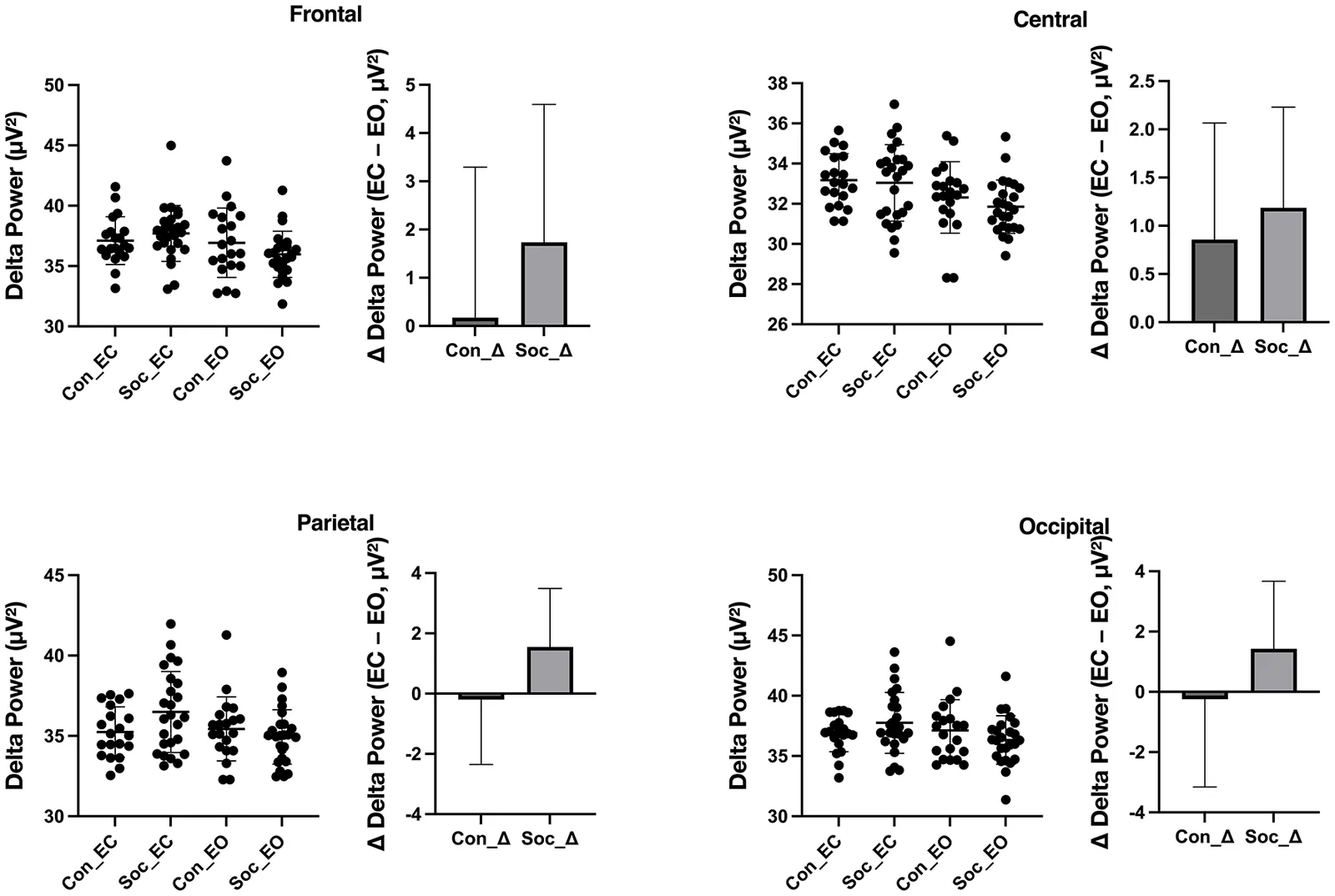
Regional patterns of delta-band EEG power during eyes-closed (EC) and eyes-open (EO) conditions and corresponding EC–EO modulation (Δ = EC – EO) in control and soccer groups.

## 4 Discussion

This study systematically compared resting-state EEG power characteristics across different brain regions and frequency bands between soccer players and non-athlete university students under eyes-open (EO) and eyes-closed (EC) conditions. The results demonstrated significant main effects of Condition and ROI across all frequency bands, indicating that the EC–EO transition was associated with reliable modulation of resting-state EEG activity under the present experimental conditions. Further analyses revealed frequency-specific patterns of group-related neural modulation, primarily involving the beta and delta bands. Specifically, the beta band exhibited a significant Condition × ROI × Group interaction, indicating differences in the spatial distribution of EEG modulation between groups during the EC–EO transition. In contrast, the delta band showed significant Condition × Group and three-way interaction effects, suggesting potentially distinct patterns of neural regulation between soccer players and non-athlete university students. *Post hoc* analyses further indicated that the EC–EO transition produced significant within-group modulation patterns across multiple regions in the beta and delta bands among soccer players, whereas the modulation patterns observed in non-athlete university students were more region-specific. However, exploratory between-group comparisons based on Δ values (EC – EO) did not remain significant after false discovery rate (FDR) correction, indicating that these regional differences should be interpreted cautiously. Collectively, these findings suggest that long-term soccer participation may be associated with selective differences in resting-state neural modulation patterns, particularly in oscillatory activity involved in the regulation of transitions between internally oriented and externally driven states.

This interpretation is consistent with classical EC/EO research, which has demonstrated that transitions between eyes-closed and eyes-open states influence not only EEG power magnitude but also its spatial distribution, with particularly pronounced effects in posterior visual-related cortical regions. Previous studies have demonstrated that EC and EO conditions differ in both EEG power and spatial organization across multiple frequency bands. Marx and colleagues reported distinct cortical activation patterns between EO and EC resting states (Marx et al., 2004), whereas Barry and colleagues further demonstrated systematic differences in power and topographical distribution across the delta, theta, alpha, and beta frequency bands (Barry et al., 2007). Petro and colleagues further demonstrated that EO conditions suppress alpha activity relative to EC, whereas other frequency bands may exhibit different directional changes, suggesting that the EC–EO transition fundamentally reflects a reorganization from an internally oriented resting state to a state characterized by external visual input (Petro et al., 2022).

First, although significant main effects of Condition and ROI, as well as significant Condition × ROI interactions, were observed in the alpha and theta bands, no Group-related effects were detected. These findings indicate that both groups exhibited similar neurophysiological responses to the classical resting-state EC–EO transition. In particular, alpha activity was significantly enhanced during the EC condition and was predominantly distributed in posterior brain regions, a pattern that is highly consistent with the established functional role of alpha oscillations in visual inhibition and internally directed attention. Previous studies have demonstrated that reduced visual input during EC leads to increased alpha activity in occipital and parietal regions, whereas EO is accompanied by visual information intake and alpha suppression (Barry et al., 2007; Klimesch, 2012; Petro et al., 2022).

Therefore, the absence of significant group-related effects in the alpha and theta bands in the present study likely reflects the stability of fundamental neurophysiological mechanisms associated with EC–EO transitions rather than specific sport-related modulation. This finding is consistent with previous research demonstrating that EC–EO transitions produce highly reproducible effects in the alpha band, which generally do not manifest as substantial differences between athlete and non-athlete populations (Petro et al., 2022). Similarly, Ramyarangsi and colleagues, when comparing EC and EO conditions across athletes from different sports, emphasized that alpha-band alterations primarily reflect visual attention and state-transition processes rather than necessarily producing a clear athlete–non-athlete distinction (Ramyarangsi et al., 2024). However, recent evidence suggests that resting-state EEG modulation during EC–EO transitions may also be influenced by methodological factors, including body posture. Specifically, posture has been shown to affect EEG spectral characteristics, particularly alpha-band activity during resting conditions (Ivaldi et al., 2023, 2026). Although posture was standardized in the present study, its potential contribution to alpha-band modulation cannot be completely excluded and should be carefully considered in future EC–EO EEG studies.

Second, the findings in the beta band revealed one of the major patterns of group-related neural modulation observed in the present study. The repeated-measures ANOVA demonstrated a significant Condition × ROI × Group interaction, indicating that the spatial distribution of EEG modulation during the EC–EO transition differed between soccer players and non-athlete university students. Further *post hoc* analyses showed that the soccer group demonstrated significant overall EC–EO modulation, whereas the control group did not show a significant overall condition effect. These findings indicate different within-group response patterns rather than definitive evidence of superior neural modulation in soccer players. The moderate effect sizes observed in some regional comparisons further suggest that future studies with larger samples may help determine whether these patterns represent reliable characteristics associated with athletic expertise. However, these findings should be interpreted in the context of the exploratory nature of the frequency-specific analyses and the lack of correction across frequency bands. Moreover, the regional modulation patterns were primarily distributed across the central, parietal, and occipital regions.

The absence of significant frontal effects in the present study should also be interpreted within the context of the EC–EO paradigm. Although frontal regions play essential roles in executive control, decision-making, and higher-order cognitive regulation, the EC–EO transition primarily reflects the shift from an internally oriented resting state to an externally driven perceptual state (Barry and De Blasio, 2017). This transition mainly involves the integration of external visual input, visuospatial orientation, and sensorimotor preparation, processes that are largely supported by posterior visual–spatial networks and sensorimotor-related cortical regions rather than frontal executive networks (Vossel et al., 2014; Wang et al., 2020). Therefore, the absence of significant frontal modulation does not necessarily indicate a lack of sport-related neural characteristics but may instead suggest that the neural processes captured by the resting-state EC–EO paradigm are more strongly associated with sensory integration and environmental monitoring networks.

From a functional perspective, beta oscillations are closely associated with active information processing, sensorimotor integration, and higher-order cognitive control processes (Engel and Fries, 2010; Kilavik et al., 2013; Spitzer and Haegens, 2017). During EO conditions, individuals are required to continuously process external visual information and monitor environmental changes. Therefore, regional alterations in beta activity may reflect changes in neural regulation involving visual–attentional–sensorimotor networks during transitions between resting states and externally oriented conditions (Kilavik et al., 2013). Barry and colleagues previously demonstrated that beta-band differences between EC and EO conditions exhibit distinct topographical characteristics, suggesting that these changes may involve localized cortical responses associated with transitions to externally oriented states. However, the contribution of general arousal-related factors cannot be completely excluded (Barry et al., 2007; Ivaldi et al., 2023, 2026). Similarly, Ramyarangsi et al. (2024) reported sport-specific regional differences in alpha, beta, and slow-wave activity under different resting-state conditions in soccer players, linking these patterns to visual attentional demands and cortical adaptation.

From the perspective of soccer-specific performance demands, the beta-band modulation pattern observed in the present study may be related to neural processes that are relevant to the visuospatial and sensorimotor demands commonly encountered during soccer participation. Soccer is a representative open-skill sport in which athletes must continuously perform large-scale visual scanning and update spatial representations based on the positions and movements of teammates, opponents, and the ball, allowing them to make tactical judgments and select appropriate motor responses within extremely limited time periods (Ashford et al., 2021). Furthermore, both training and competition require athletes to continuously monitor external environmental information and rapidly integrate visual perception, spatial orientation, and motor preparation under highly dynamic and complex conditions (Vestberg et al., 2012). Consequently, when transitioning from an internally oriented resting state (EC) to an externally oriented environmental monitoring state (EO), soccer players may exhibit different regional patterns of EEG modulation involving central–parietal–occipital regions. Functionally, the central region is primarily involved in sensorimotor integration and motor preparation, the parietal region plays an essential role in spatial attention and visuospatial processing, whereas the occipital region is mainly responsible for visual information processing (Corbetta and Shulman, 2002). Therefore, the regional beta-band EC–EO modulation pattern observed in soccer players may reflect neural characteristics associated with networks involved in visual–attentional–sensorimotor processing. However, whether these neural characteristics directly correspond to superior soccer-specific cognitive abilities remains unclear. Because the present study employed resting-state EEG without concurrent behavioral assessment, the functional significance of these neural patterns should be considered exploratory and requires validation using sport-specific cognitive tasks.

Furthermore, the beta-band findings did not indicate a generalized enhancement across the entire brain but instead demonstrated region-specific modulation. This pattern can be interpreted within the broader framework of functional specialization and neural optimization, suggesting that expertise-related neural characteristics may involve selective and functionally relevant recruitment of neural resources rather than uniform increases in cortical activity. According to this framework, expertise-related neural characteristics may involve selective patterns of neural regulation that correspond to demands associated with specific experiences (Neubauer and Fink, 2009). Previous studies have demonstrated that athletes may exhibit reduced, enhanced, or spatially focused patterns of neural activity, with the common characteristic that these patterns are closely matched to specific performance demands (Babiloni et al., 2010; Li and Smith, 2021).

For soccer players, long-term sport-specific training may be associated with selective functional adaptations in brain regions particularly relevant to performance requirements rather than generalized alterations across the entire cortex (Sultanov et al., 2026). Processes such as visual search, spatial updating, multiple-object tracking, and rapid decision-making during soccer competition involve coordination among central, parietal, and occipital networks. Therefore, the region-specific beta-band modulation observed in the present study may be related to neural processes involved in visual–attentional–sensorimotor regulation, although this interpretation remains speculative without behavioral validation.

Previous EEG studies investigating neural characteristics in athletes have largely focused on male athletes or mixed-sex samples, whereas female athletes remain comparatively underrepresented (Baumeister et al., 2008; Cheron et al., 2016). Similar to previous observations in predominantly male athlete populations, the present study identified frequency- and region-specific EEG modulation patterns rather than generalized alterations across the entire cortex. In particular, the modulation observed within beta and delta bands in posterior and sensorimotor-related regions is generally consistent with previous evidence suggesting that long-term sport participation is associated with functional characteristics in neural networks supporting visuospatial processing and sensorimotor integration (Kilavik et al., 2013; Del Percio et al., 2019). However, direct comparisons between female and male athletes remain limited. Sex-related differences in neuroplasticity, hormonal regulation, training history, and sport participation characteristics may contribute to variations in EEG characteristics associated with athletic expertise. Therefore, the present findings extend previous evidence derived largely from male or mixed-sex samples to female soccer players, while future studies directly comparing male and female athletes are needed to clarify whether sex-specific mechanisms contribute to sport-related neural adaptations.

Third, the findings in the delta band provided additional evidence regarding frequency-specific neural modulation patterns. Unlike the beta band, the delta band exhibited significant Condition × Group interaction effects, indicating that the EC–EO transition was modulated differently between soccer players and non-athlete university students. Furthermore, estimated marginal means (EMMs) revealed significant EC–EO differences across all ROIs in the soccer group, whereas the control group demonstrated a more localized modulation pattern restricted to the central region. These findings suggest that soccer players exhibited potentially different patterns of neural regulation during transitions between internally and externally oriented states. However, the extent to which these patterns represent sport-specific adaptations requires further investigation. However, exploratory between-group comparisons based on Δ values did not remain significant after FDR correction, and therefore the regional differences identified in the present study should be interpreted cautiously. Nevertheless, the effect size estimates suggest that some regional comparisons may represent potentially meaningful differences, although the wide confidence intervals indicate considerable uncertainty and highlight the need for replication in larger samples. Although delta oscillations have traditionally been classified as slow-wave activity, accumulating evidence indicates that delta activity during wakeful resting states does not simply reflect reduced arousal but may also contribute to attentional resource allocation and cognitive control processes (Başar et al., 2000; Knyazev, 2012; Harmony, 2013). Considering the important roles of the parietal and occipital regions in spatial attention and visual processing, the present findings may indicate that soccer players show different patterns of delta-band modulation in posterior regions, although the relationship between these EEG patterns and visuospatial processing requires further investigation.

Notably, the observed group-related modulation patterns were not distributed across all frequency bands but were primarily observed in the beta and delta bands. This frequency-specific pattern is consistent with the functional specialization of different EEG oscillations and with previous evidence suggesting that sport-related neural characteristics may preferentially involve frequency bands associated with active information processing, environmental adaptation, and sensorimotor integration. As a representative open-skill sport, soccer requires athletes to continuously integrate external visual information and rapidly generate appropriate responses within highly dynamic and unpredictable environments. These processes rely heavily on complex perceptual–cognitive processing and executive functions (Vestberg et al., 2017; Lai et al., 2024). Therefore, long-term soccer participation may be associated with selective modulation of neural oscillations involved in environmental information processing and sensorimotor integration rather than broad alterations in resting-state EEG activity itself. This interpretation is also consistent with the broader literature on sport-related brain oscillations, which suggests that different frequency bands serve distinct functional roles, with alpha activity primarily associated with inhibitory mechanisms, whereas beta and delta activity are more closely related to active information processing and adaptation to environmental demands (Cheron et al., 2016; Li and Smith, 2021).

From a practical perspective, the present findings have several potential implications. First, the EC–EO paradigm represents a low-demand and easily standardized resting-state assessment approach that may provide sensitive information regarding differences in neural regulatory characteristics between athletes and non-athletes, particularly in frequency bands involved in active environmental processing. Second, the regional modulation patterns observed in posterior and sensorimotor-related regions suggest that soccer-related neural characteristics may involve networks that have been implicated in visuospatial processing and sensorimotor regulation. However, behavioral validation is required before these EEG characteristics can be interpreted as indicators of sport-specific cognitive advantages. Third, compared with absolute power measures obtained under a single resting-state condition, EC–EO modulation may better capture the dynamic capacity of the nervous system to adapt between internally oriented and externally driven states, and therefore may represent a potential approach for characterizing neural regulation in athletic populations (Büchel et al., 2021; Ramyarangsi et al., 2024; Shi et al., 2024).

Several limitations of the present study should be acknowledged. First, although significant interaction effects and group-related modulation patterns were identified in the beta and delta bands, the present study analyzed frequency bands separately based on their distinct physiological characteristics. This approach may increase the number of statistical comparisons across frequency-specific analyses. Although exploratory between-group comparisons of Δ values (EC – EO) were additionally controlled using false discovery rate (FDR) correction, the repeated-measures ANOVA models were conducted separately for each frequency band and were not subjected to an additional correction across the four frequency bands. Therefore, the Condition × ROI × Group interaction effects observed in the beta and delta bands should be considered exploratory and interpreted cautiously. Future studies using larger samples and preregistered analytical strategies are needed to further validate these frequency-specific neural modulation patterns.

Second, the cross-sectional design of the present study limits causal interpretation regarding whether the observed EEG characteristics were induced by long-term soccer training or reflected pre-existing differences between athletes and non-athletes. Although all soccer players had extensive systematic training experience and competitive backgrounds, detailed information regarding current training volume, training intensity, and cardiovascular fitness level was not systematically quantified. These factors may represent potential sources of individual variability in resting-state EEG characteristics and should be considered in future studies investigating neural characteristics associated with athletic expertise.

Third, several methodological factors related to EEG acquisition and experimental procedures should be considered. The order of resting-state conditions was fixed, with the eyes-open (EO) condition always preceding the eyes-closed (EC) condition. Although this procedure ensured a consistent experimental protocol across participants, potential order-related effects, such as habituation, vigilance fluctuation, or temporal changes in alertness, cannot be completely excluded. In addition, although all EEG recordings were conducted under standardized laboratory conditions and participants maintained a consistent seated posture throughout the resting-state assessment, the potential influence of body posture was not experimentally manipulated or statistically examined. Recent evidence suggests that body posture may influence resting-state EEG characteristics, particularly alpha-band modulation during eyes-closed and eyes-open transitions (Ivaldi et al., 2023, 2026). Furthermore, the number of electrodes included in each ROI was not identical, particularly for the occipital region, which contained only two electrodes (O1 and O2). Although this ROI definition followed the conventional 10–20 system and was consistent with previous EEG studies, the relatively limited electrode coverage may influence regional estimates and should be considered when interpreting ROI-related effects. Future studies should adopt randomized or counterbalanced condition orders, systematically investigate posture-related influences, and employ higher-density EEG recordings to provide a more comprehensive characterization of regional neural modulation patterns.

Fourth, the present study employed a resting-state EEG paradigm, and the observed neural characteristics primarily reflect fundamental state-transition processes rather than online cognitive processing during sport-specific tasks or competitive situations. In addition, the absence of behavioral measurements limits direct interpretation regarding whether the observed EEG modulation is associated with differences in visuospatial attention, decision-making ability, or soccer-specific performance. Although the observed EEG patterns may be related to neural processes involved in environmental information processing and sensorimotor regulation, alternative explanations such as differences in general arousal, cardiovascular fitness, and training-related characteristics cannot be completely excluded. Therefore, the functional interpretation of beta- and delta-band modulation should be regarded as hypothesis-generating rather than definitive evidence of sport-specific cognitive adaptations. Future studies combining resting-state EEG with behavioral assessments, physiological measurements, and sport-specific cognitive tasks are needed to further clarify the functional significance of these neural patterns.

Finally, although the present study focused on female soccer players, sex-related influences on neural adaptation remain insufficiently understood. Direct comparisons between male and female athletes are required to determine whether sex-specific mechanisms contribute to sport-related neural characteristics.

## 5 Conclusion

The present study systematically compared resting-state EEG power characteristics across different brain regions and frequency bands between female soccer players and non-athlete female university students under eyes-open (EO) and eyes-closed (EC) conditions. The findings demonstrated that the EC–EO transition reliably modulated resting-state EEG activity, as reflected by significant effects of Condition and ROI across frequency bands.

Further analyses revealed frequency-specific patterns of group-related neural modulation, particularly in the beta and delta bands. The beta band exhibited a significant Condition × ROI × Group interaction, indicating that the spatial distribution of EC–EO modulation differed between groups. Similarly, the delta band showed significant Condition × Group interaction effects, suggesting different patterns of EEG modulation during transitions between resting states in soccer players and non-athlete university students. *Post hoc* analyses further indicated distinct regional EC–EO modulation patterns within the beta and delta bands. However, exploratory between-group comparisons of Δ values (EC – EO) did not remain significant after false discovery rate correction, suggesting that these regional differences should be interpreted cautiously.

In contrast, the alpha and theta bands showed no significant group-related effects, suggesting that these frequency bands primarily reflected fundamental neurophysiological responses associated with the transition between internally oriented resting states and externally driven visual states rather than clear sport-specific modulation.

Overall, long-term soccer participation may be associated with selective differences in resting-state neural regulatory patterns involving oscillatory processes related to environmental monitoring and sensorimotor regulation, rather than generalized alterations in resting-state neural oscillatory characteristics. These findings extend current knowledge regarding EEG characteristics in female soccer players and highlight the potential value of the EC–EO paradigm for investigating dynamic neural regulation in athletic populations. Future studies combining resting-state EEG with behavioral and sport-specific cognitive assessments are warranted to further clarify the functional significance of these neural patterns.

## Data Availability

The raw data supporting the conclusions of this article will be made available by the authors, without undue reservation.
